# Partial-body cryostimulation after training improves sleep quality in professional soccer players

**DOI:** 10.1186/s13104-019-4172-9

**Published:** 2019-03-15

**Authors:** Wafa Douzi, Olivier Dupuy, Dimitri Theurot, Geoffroy Boucard, Benoit Dugué

**Affiliations:** 1 Laboratoire Mobilité Vieillissement Exercice (MOVE)-EA6314, Faculty of Sport Sciences, University of Poitiers, 8 allée Jean Monnet, 86000 Poitiers, France; 2 Centre de Recherches sur la Cognition et l’Apprentissage (UMR7295), Université de Poitiers and Université François-Rabelais de Tours, Poitiers, France

**Keywords:** Cryostimulation, Cryotherapy, Exercise recovery, Sleep, Soccer

## Abstract

**Objective:**

The aim of the present investigation was to determine whether using cryostimulation (partial-body cryostimulation) impacts sleep quality in professional soccer players. Different exposure durations at − 180 °C were tested randomly after standardized training sessions in nine professional soccer players (no cryostimulation, 180-s exposure, two 90-s exposures separated by a 5-min rest at room temperature, and 90-s exposure), and the effects on sleep quality using 3-dimensional accelerometers worn during sleep were assessed.

**Results:**

The number of movements during the night after partial-body cryostimulation was significantly reduced only in the 180-s exposure condition (p < 0.05, very large effect size) compared with the control condition. Partial-body cryostimulation seems to induce a positive impact on sleep quality that may be dose-dependent.

*Trial registration* Australia and New Zealand Clinical Trials Registry (ANZCTR), ACTRN12619000330145, date of registration: 4/03/2019. Retrospectively registered

## Introduction

Currently, cryostimulation is commonly used in athletes for recovery purposes after physical exercise and consists of short exposure (usually 3 min) in minimal clothing at very low temperature (− 110 °C to − 180 °C) in a special chamber. Two types of cryochambers are available: whole-body and partial-body (the head is not exposed) cryochambers. The benefits of being exposed at low temperature concern pain, fatigue, delayed onset of muscle soreness, oxidative stress, inflammation and a better recovery after exercise [1, 2]. As a short term cold exposure may induce a stronger parasympathetic tone, and considering that the parasympathetic control of heart rate is predominant during the non-rapid eye movement sleep (that includes deep sleep period) [3], it has been hypothesized that cold stimulation (ice-cold bath and cryostimulation) after exercise could have a positive effect on sleep quality [4–7]. A reduction in muscle soreness after the cold exposure has also been proposed to influence the quality of sleep [2].

In the context of recovery, sleep is clearly essential, both for cognitive processes and metabolic functions. A recent review explored the links between stress, sleep and recovery in elite soccer players [8]. During the different sleep stages, tissue regeneration may occur through the elimination of neurotoxic wastes, and a good sleep quality promotes the recovery of cognitive functions [9]. A decrease in psychomotor functions has been observed already after a single night of reduced sleep [10]. An earlier study demonstrated that an increase in the training volume in elite swimmers led to poorer sleep quality as inferred from an increase in the number of movements during sleep [11]. However, there is presently a lack of information concerning the link between cold exposure after exercise and its impact on the quality of sleep. Nevertheless, a recent work showed that a 3-min whole body cryotherapy/cryostimulation after training in the evening improves sleep quality in physically active men [12]. Additionally, data collected from competitive athletes are missing. It seems that there are only two preliminary studies on that topic, both showing an improvement in the quality of sleep in ten elite synchronized female swimmers [4] and in 27 professional basketball players [5]. In these two reports, a 3-min exposure was investigated because such a duration was commonly used in sport activities for recovery purposes. To date, the exposure duration has never been studied, and in the context of sleep recovery, a dose–response effect may be possible.

The aim of the present investigation was, therefore, to determine whether the use of cryostimulation (partial-body cryostimulation) has an impact on sleep quality in professional soccer players. Different exposure durations at − 180 °C were tested randomly after standardized training sessions (no cryostimulation, 180-s exposure, twice 90-s exposures separated by a 5-min rest at room temperature, and 90-s exposure), and the effects on sleep quality were studied using 3-dimensional accelerometers worn during sleep.

## Main text

### Methods

#### Subjects

Nine male football (soccer) professional players (24.8 ± 5.5 years; 76.7 ± 7.3 kg; 184 ± 0.1 cm) from the Niort Football Club (French National Championship Ligue 2) participated in the study. After medical examination and receipt of informed consent, the subjects were enrolled in the investigation which conformed to the Code of Ethics of the World Medical Association (Declaration of Helsinki), and were accepted by ethics committee (CERSTAPS n°2018-12-07-25).

#### Experimental design

The experiment was organized during 1 month, and each session took place once a week on the same week day and at the same time during the day (12:00 p.m.). Each player undertook a standardized 90-min training session which consisted in 10 min warm-up, 30 min of technical and tactical work, 30 min of interval running (15-s at 95% of the player maximal aerobic speed followed by 15-s of passive recovery), and 20 min of plyometric training. The training session was conducted under the supervision of the head coach and the physical trainer. After the training session, each player experienced different exposure durations at − 180 °C in a partial-body chamber (Cryotechno^®^, Castelnau le Lez, France) in a random order (no cryostimulation, 180-s exposure, twice 90-s exposure separated by a 5-min rest at room temperature, and 90-s exposure). A partial-body chamber consists of an open tank in which the subject is exposed to cold, excluding the head and neck [1]. The cold temperature is obtained by spraying nitrogen (expanded nitrogen) inside the tank. Such devices have already been described [1, 5, 7]. In the cabin, the subjects wore bathing suits, a pair of gloves, socks, and slippers. The subjects were instructed to turn around continuously in the cabin during the exposure period. The cryostimulation sessions were undertaken under medical supervision. During the time of the experiment, the players had a 90-min training session daily six times per week and one official match.

#### Measurements

##### Heart rate variability (HRV)

The HRV was assessed before and after training sessions using heart rate monitors (Polar V800 GPS, Finland) to assure that the training load was similar in both sessions according to Kaikkonen et al. [13].

##### Temperature measurements and estimations

The surface temperature of quadriceps was measured using an infrared thermometer after the subjects left the cryo device. Additionally, the subjects were asked to rate their perceptual thermal sensation using a 10-point scale [14]. According to their response to the question “How cold do you feel right now?”, the subjects were scored from 0 (“neutral”) to 10 (“unbearably cold”).

##### Sleep quality assessment using accelerometry

Every night after cryotherapy exposure or control session, the subjects were instructed to wear a wrist actigraph (WGT3X-BT monitor, Pensacola, USA) to monitor their sleep patterns [15]. They were instructed to behave similarly each time their sleep was monitored. The recording was manually started by the subjects at the time they went to bed, and the recording was stopped when they woke up. The accelerometer measured movement acceleration across the horizontal, vertical, and perpendicular axes. The raw data were recorded with an epoch length of 60 s and were extracted as the sum of vector magnitude in counts/min (calculated as the square root of the sum of the square of acceleration for each of the three axes) using actiLife software (version 6.11.0, Fort Walton Beach, FL, USA). Sleep efficiency was calculated as the ratio of the “actual sleep time” divided by the total sleep time multiplied by 100, where the “actual sleep time” was the total sleep time minus the wake time. The wake time was calculated as the number of min where the number of movements were over the threshold of 40 counts/min [16].

##### Subjective sleep quality

The sleep quality was evaluated using a Spiegel Sleep Quality Perception Questionnaire [17].

#### Statistics

The results are expressed as mean and standard deviation (SD) values or standard error when specified. The Gaussian distribution was tested for each variable using the Shapiro–Wilk test. Changes in the different variables were evaluated using repeated-measures ANOVA followed by Tukey post hoc test when appropriate. The effect size of the changes was assessed by the Hedges’ g (g) as presented by Dupuy et al. [18] and was considered to be either small (0.2 < g ≤ 0.5), moderate (0.5 < g ≤ 0.8), or large (g > 0.8) according to the Cohen scale [19]. A p < 0.05 was considered to be statistically significant. The required sample size was calculated from our control data concerning the number movements (counts/min) during the sleeping time using G*Power version 3.1, according to Beck [20]. Using an a priori repeated-measures design with a desired power (1-beta) set at 0.80, and an alpha risk of 0.05, six subjects represent a sufficient number of subjects to detect a significant difference.

### Results

**Fig. 1:**
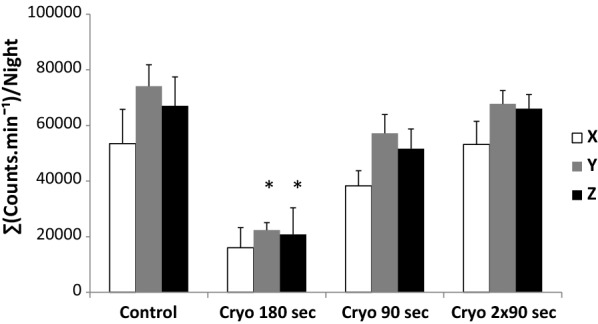
The sum of the counts per minute on the 3 movement axes (x, horizontal; y, vertical; and z, perpendicular) during the night following cryostimulation exposure (180 s, 90 s, and 2 × 90 s) and the control session. *Significant difference from the control at p < 0.01. The data are expressed as the mean ± SE values

**Fig. 2:**
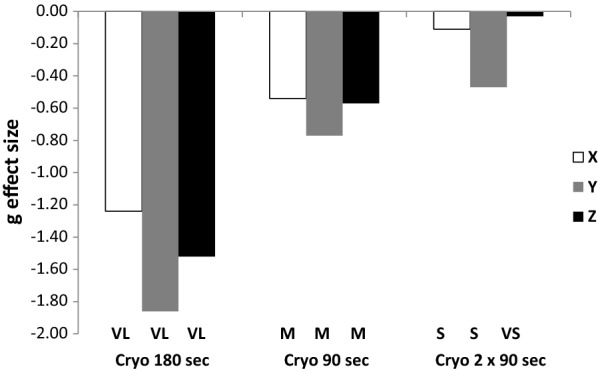
Effect size (Hedges’s g) of changes in activity counts in three spatial axes (x, horizontal; y, vertical; and z, perpendicular) during the night following partial-body cryostimulation exposures (180 s, 90 s, and 2 × 90 s) compared with the control session. VL, very large effect size; M, medium effect size; S, small effect size; VS, very small effect size

Exposure at − 180 °C led to a significant decrease (p < 0.001) in skin temperature at the quadriceps level. The highest decrease was seen at the 180-s exposure (from 30.4 ± 1.0 to 15.4 ± 2.0 °C) which was significantly higher (p < 0.001) than similar decreases observed for the 90-s and the 2 × 90-s exposures (from 29.6 ± 1.0 °C to 20.7 ± 1.6 °C and from 29.3 ± 1.4 to 19.3 ± 1.0 °C, respectively). Similarly, the perception of cold was significantly higher (p < 0.001) after the 180-s exposure (8.0 ± 1.2) than after the 90-s and the 2 × 90-s exposures (4.6 ± 1.8 and 5.3 ± 1.2, respectively). The night following the exposure, the number of accelerations detected on the vertical and perpendicular axes during sleep were significantly lower after the 180-s exposure than in the control and in other tested conditions (p values ranged from 0.01 to 0.05) (Fig. 1). The size of the effects was considered to be very large after the 180-s exposure (g > − 1.24) (Fig. 2). The sleep efficiency tended to be higher after the 180-s exposure than after the other exposures but the statistical significance was not reached (p < 0.09). The subjective sleep quality was similar in all the tested conditions. It has to be noted that two subjects did not kept the wrist actigraph throughout the entire night and their data were excluded from this analysis. Concerning the HRV data obtained before and after the training sessions, a significant main effect (p < 0.05) was observed between the two sessions concerning mean heart rate, high-frequency (HF) band power, low-frequency (LF) band power, LF/HF power ratio. However, no significant interactions were noted between the different tested sessions.

### Discussion

The main finding in this study is that the use of partial-body cryostimulation after training improves the night sleep quality in professional soccer players. This improvement in sleep quality was deduced from a significant reduction in the number of movements during sleep [15]. We used 3-dimensional accelerometer-based activity monitors (Actigraphs) to detect this movement reduction. Previous studies have shown that the actigraphic technique is validated and reliable for measuring sleep quality and provides insight into sleep–wake patterns [21–24]. During sleep, low motor activity levels and prolonged episodes of uninterrupted immobility are associated with increasing sleep depth, whereas high activity levels are related to intermittent wakefulness during sleep [25]. Interestingly, a decrease in the number and frequency of rotational motions (rollovers) depicted from a perpendicular axis seems linked to an increase in deep sleep [23]. The deep sleep stage is considered essential for recovering from mental and physical fatigue. Thus, a decrease in the number of movements—especially those from the perpendicular axis—in the night following partial-body cryostimulation may be related to an increase in a deeper sleep in our subjects. Recently, several attempts to evaluate the effect of slightly decreasing body temperature on sleep quality have been performed. Cold immersion near bedtime have been shown to improve sleep propensity by accelerating the decline rate in human internal temperature [26]. Al Haddad et al. found that a 5-min immersion in cold water (15 °C) following daily training improved subjective sleep quality in athletes [6].

Our results are consistent with these observations and strengthen two preliminary studies on sleep quality after cryostimulation in competitive athletes [4, 5]. The first study showed a positive effect in a series of 14 3-min whole-body cryostimulation (WBC) exposures at − 110 °C (one per day for 14 consecutive days) on sleep latency and efficiency (estimated using 3-dimensional accelerometers) in ten elite synchronized female swimmers during an intense training period leading to overreaching [4]. The cold exposure improved sleep quality and promoted relaxation and the onset of sleepiness, especially during phases of increased workload [4].

In the second study, 27 elite basketball players reported an enhancement in their sleep quality perception during international competitions when a 3-min partial-body cryostimulation exposure at − 130 °C was used [5]. In these two investigations, a 3-min exposure was investigated. The present work is the first to compare different cold exposure durations on sleep quality in professional athletes after similar and standardized training sessions (the training load and subsequent fatigue were also indirectly checked using heart rate variability which was found to be similar in all tested situations). Among the durations we tested, the 180-s exposure at − 180 °C induced an improvement in the sleep quality in our subjects. The other durations (90 s and 2 × 90 s) were unable to induce such a change. The cold stimulation in these latter exposures was certainly not strong enough as observed in the relatively modest decrease in skin temperature (approximately − 10 °C), whereas the 180-s exposure induced a larger decrease (approximately − 15 °C). Therefore, in the context of partial-body cryostimulation after training and sleep recovery, a dose–response effect may be possible.

It seems that a sufficient decrease in skin temperature is necessary to induce an improvement in sleep quality. The mechanisms involved in such feature can be related to the analgesic effect of the cold stimulation which lowered the exercise-induced muscle soreness in our professional athletes facilitating therefore relaxation and onset of sleepiness, diminishing arousal in the evening and the number of awakenings during the night and finally improving sleep quality. Beta-endorphin and noradrenalin (neurotransmitters exerting analgesic effects) have been shown to be produced after cryostimulation [27]. Also, cryostimulation has been shown to lead to a reactivation of parasympathetic nervous activity and an increase in the parasympathetic activity has been linked to a better sleep quality [4, 28]. In our recent work with physically active men, a 3-min whole-body cryostimulation (− 40 °C, wind speed of 2.3 m/s) after training in the evening was able to induced a better sleep quality in our volunteers. Interestingly, we were able to detect an increase in the parasympathetic tone during the slow wave sleep episodes (deep sleep) which was connected with fewer body movements and a better sleep quality [12]. Even though the cryostimulation was long before bed time in the present investigation regarding professional athletes, and even though the experimental setting was different, a similar feature might have appeared. Nevertheless, the physiological mechanisms underlying such improvements remain to be elucidated.

In conclusion, the use of partial-body cryostimulation (180-s at − 180 °C) after training improves sleep quality in professional soccer players.

### Limitations

This work is a small scale trial and should be completed to assure that sleep quality is improved by using partial-body cryostimulation in athletes after physical exercise.

## References

[1] Bouzigon R, Grappe F, Ravier G, Dugué B (2016). Whole- and partial-body cryostimulation/cryotherapy: current technologies and practical applications. J Therm Biol.

[2] Dupuy O, Douzi W, Theurot D, Bosquet L, Dugué B (2018). An evidence-based approach for choosing post-exercise recovery techniques to reduce markers of muscle damage, soreness, fatigue and inflammation: a systematic review with meta-analyses. Front Physiol.

[3] Stein P, Pu Y (2012). Heart rate variability, sleep and sleep disorders. Sleep Med Rev.

[4] Schaal K, Le Meur Y, Louis J, Filliard J-R, Hellard P, Casazza G (2015). Whole-body cryostimulation limits overreaching in elite synchronized swimmers. Med Sci Sports Exerc.

[5] Bouzigon R, Ravier G, Dugué B, Grappe F (2014). The use of whole-body cryostimulation to improve the quality of sleep in athletes during high level standard competitions. Br J Sports Med.

[6] Al Haddad H, Parouty J, Buchheit M (2012). Effect of daily cold water immersion on heart rate variability and subjective ratings of well-being in highly trained swimmers. Int J Sports Physiol Perform.

[7] Louis J, Schaal K, Bieuzen F, Le Meur Y, Filliard J, Volondat M (2015). Head exposure to cold during whole-body cryostimulation: influence on thermal response and autonomic modulation. PLoS ONE.

[8] Nédélec M, Halson S, Abaidia A, Ahmaidi S, Dupont G (2015). Stress, sleep and recovery in elite soccer: a critical review of the literature. Sports Med.

[9] Martin J, Song Y, Hughes J, Jouldjian S, Dzierzewski J, Fung C (2017). A four-session sleep intervention program improves sleep for older adult day health care participants: results of a randomized controlled trial. Sleep.

[10] Reilly T, Edwards B (2007). Altered sleep–wake cycles and physical performance in athletes. Physiol Behav.

[11] Taylor S, Rogers G, Driver H (1997). Effects of training volume on sleep, psychological, and selected physiological profiles of elite female swimmers. Med Sci Sports Exerc.

[12] Douzi W, Dupuy O, Tanneau M, Boucard G, Bouzigon R, Dugué B (2019). 3-min whole body cryotherapy/cryostimulation after training in the evening improves sleep quality in physically active men. Eur J Sport Sci..

[13] Kaikkonen P, Hynynen E, Mann T, Rusko H, Nummela A (2010). Can HRV be used to evaluate training load in constant load exercises?. Eur J Appl Physiol.

[14] Lundgren P, Henriksson O, Kuklane K, Holmér I, Naredi P, Björnstig U (2014). Validity and reliability of the Cold Discomfort Scale: a subjective judgement scale for the assessment of patient thermal state in a cold environment. J Clin Monit Comput.

[15] Mullaney D, Kripke D, Messin S (1980). Wrist-actigraphic estimation of sleep time. Sleep.

[16] Sargent C, Lastella M, Halson S, Roach G (2016). The validity of activity monitors for measuring sleep in elite athletes. J Sci Med Sport..

[17] Spiegel R (1984). Sleep disorders in the aged. Internist..

[18] Dupuy O, Lussier M, Fraser S, Bherer L, Audiffren M, Bosquet L (2014). Effect of overreaching on cognitive performance and related cardiac autonomic control. Scand J Med Sci Sports.

[19] Cohen J (1988). Statistical power analysis for the behavioral sciences.

[20] Beck T (2013). The importance of a priori sample size estimation in strength and conditioning research. J Strength Cond Res..

[21] Kaartinen J, Erkinjutti M, Rauhala E (1996). Automatic SCSB analysis of motor and autonomic nervous functions compared with sleep stages. NeuroReport.

[22] Kushida C, Chang A, Gadkary C, Guilleminault C, Carrillo O, Dement W (2001). Comparison of actigraphic, polysomnographic, and subjective assessment of sleep parameters in sleep-disordered patients. Sleep Med.

[23] Miwa H, Sasahara S, Matsui T (2007). Roll-over detection and sleep quality measurement using a wearable sensor. Conf Proc IEEE Eng Med Biol Soc..

[24] Wang D, Wong K, Dungan G, Buchanan P, Yee B, Grunstein R (2008). The validity of wrist actimetry assessment of sleep with and without sleep apnea. J Clin Sleep Med.

[25] Middelkoop H, Van Hilten B, Kramer C, Kamphuisen H (1993). Actigraphically recorded motor activity and immobility across sleep cycles and stages in healthy male subjects. J Sleep Res.

[26] Kräuchi K (2007). The thermophysiological cascade leading to sleep initiation in relation to phase of entrainment. Sleep Med Rev.

[27] Leppäluoto J, Westerlund T, Huttunen P, Oksa J, Smolander J, Dugué B (2008). Effects of long-term whole-body cold exposures on plasma concentrations of ACTH, beta endorphin, cortisol, catecholamines and cytokines in female subjects. Scand J Clin Lab Invest.

[28] Schaal K, Le Meur Y, Bieuzen F, Petit O, Hellard P, Toussaint J-F (2012). Effect of recovery mode on postexercise vagal reactivation in elite synchronized swimmers. Appl Physiol Nutr Metab.

